# Myo/Nog cells are nonprofessional phagocytes

**DOI:** 10.1371/journal.pone.0235898

**Published:** 2020-08-24

**Authors:** Jacquelyn Gerhart, Lindsay Gugerty, Paul Lecker, Fathma Abdalla, Mark Martin, Olivia Gerhart, Colby Gerhart, Karanveer Johal, Jake Bernstein, John Spikes, Keith Mathers, Arturo Bravo-Nuevo, Mindy George-Weinstein

**Affiliations:** 1 Philadelphia College of Osteopathic Medicine, Philadelphia, Pennsylvania, United States of America; 2 Main Line Surgery Center, Bala Cynwyd, Pennsylvania, United States of America; Washington State University, UNITED STATES

## Abstract

Myo/Nog cells were discovered in the chick embryo epiblast. Their expression of MyoD reflects a commitment to the skeletal muscle lineage and capacity to differentiate into myofibroblasts. Release of Noggin by Myo/Nog cells is essential for normal morphogenesis. Myo/Nog cells rapidly respond to wounding in the skin and eyes. In this report, we present evidence suggesting that Myo/Nog cells phagocytose tattoo ink in tissue sections of human skin and engulf cell corpses in cultures of anterior human lens tissue and magnetic beads injected into the anterior chamber of mice *in vivo*. Myo/Nog cells are distinct from macrophages in the skin and eyes indicated by the absence of labeling with an antibody to ionized calcium binding adaptor molecule 1. In addition to their primary roles as regulators of BMP signaling and progenitors of myofibroblasts, Myo/Nog cells behave as nonprofessional phagocytes defined as cells whose primary functions are unrelated to phagocytosis but are capable of engulfment.

## Introduction

Myo/Nog cells were discovered in the chick embryo blastocyst by their expression of the skeletal muscle specific transcription factor MyoD and Noggin, an inhibitor of bone morphogenetic proteins [1, 2]. The subpopulation of approximately 10 Myo/Nog cells in the epiblast expands as they become incorporated into all three germ layers [3, 4]. Later in development, they are found in low numbers in multiple organ systems [5]. Myo/Nog cells continue to express MyoD mRNA regardless of their environment and are refractive to inductive signals that specify commitment to non-skeletal muscle lineages [4].

While Myo/Nog cells are capable of forming myofibers *in vitro*, their primary role during skeletal muscle formation in the early embryo is to regulate the onset of differentiation via release of the bone morphogenetic protein (BMP) inhibitor Noggin [3–6]. Depletion of Myo/Nog cells in the blastocyst results in hyperactive BMP signaling, an absence of skeletal muscle and expansion of cardiac muscle in the paraxial mesoderm [3, 7]. Embryos lacking Myo/Nog cells are grossly malformed with defects in the anterior body wall, nervous system, face and eyes [3, 7, 8].

In addition to their role as regulators of BMP signaling, Myo/Nog cells respond to injury in the embryo and adult. They rapidly migrate to areas of cell death in the epiblast [7]. In adult human and murine skin, Myo/Nog cells are normally found in a niche associated with hair follicles [9]. Within 24 hours of epidermal abrasion, they increase in number and populate the wound [9]. Similar behaviors of population expansion and homing to areas of injury were observed in the retina [10, 11]. Their depletion in the hypoxic mouse retina results in increased neuronal cell death, whereas addition of Myo/Nog cells to the vitreous reduces photoreceptor cell loss and preserves function following light damage in rats [10, 11]. Thus, Myo/Nog cells are neuroprotective in the retina.

The dynamic behavior of Myo/Nog cells also has been observed in the lens. Upon injury to the lens epithelium, they migrate to the wound and differentiate into myofibroblasts that synthesize alpha smooth muscle actin (α-SMA) and striated muscle specific proteins [12–16]. Eliminating Myo/Nog cells in explant cultures of human lens tissue with either the targeting G8 monoclonal antibody (mAb) and complement or a drug consisting of the G8 mAb conjugated to 3DNA nanocarriers for doxorubicin prevents the emergence of myofibroblasts [14]. Injection of this drug into the rabbit lens during cataract surgery significantly reduces myofibroblasts and mitigates a vision impairing, fibrotic disease called posterior capsule opacification [16].

Further evidence of Myo/Nog cells’ reactivity to disruptions in homeostasis is their presence in skin tumors and sarcomas [9, 17]. In human squamous and basal cell carcinomas and malignant melanomas, the number of Myo/Nog cells correlates with tumor stage and grade [9]. A subpopulation of Myo/Nog cells in melanomas contained pigment (9). This finding, along with the observation that Myo/Nog cells are attracted to wounds and dying cells in the skin, lens and retina [9] suggest that Myo/Nog cells may be involved in clearance of cell and tissue debris. In this study, we examined human tattooed skin, cultured human lens tissue and the anterior cavity of rabbit eyes injected with magnetic beads for evidence of Myo/Nog cell mediated phagocytosis.

## Materials and methods

### Procurement of tissues

A 6 mm punch biopsy needle was used to collect samples of tattooed skin from human bodies donated to the Philadelphia College of Osteopathic Medicine through the Humanity Gifts Registry of Pennsylvania. Tissue was embedded in paraffin and sectioned at 10 μM.

Anterior human lens tissue was removed by capsulorhexis during cataract surgery. Procurement of tissue was carried out in accordance with the Declaration of Helsinki and approved by the Philadelphia College of Osteopathic Medicine’s Institutional Review Board (#H17-018). Written consent was obtained from all patients. Tissue was collected in Dulbecco’s modified Eagle’s medium (DMEM)/F12) containing 3 IU penicillin and 30 μg streptomycin (Gibco; Thermo Fisher Scientific, Waltham, MA).

New Zealand white female rabbits weighing 2.8 to 3.2 kg underwent cataract surgery as described previously [16]. Anesthesia was obtained with an intramuscular injection of ketamine hydrochloride (50 mg/kg) and xylazine (7 mg/kg). One drop of topical proparacaine hydrochloride anesthetic was placed in each eye before surgery and all efforts were made to minimize suffering. The procedures adhered to the Association for Research in Vision and Ophthalmology Statement for the Use of Animals in Ophthalmic and Vision Research and approved by the Institutional Animal Care and Use Committee (IACUC) of the University of Utah. The eyes were harvested two days after surgery, fixed in 10% neutral buffered formalin, bisected coronally, and the anterior cavity was embedded in paraffin and sectioned at 10 μM.

Three-month old male C57BL/6J mice (Jackson Laboratory, Bar Harbor, ME) received injections of magnetic beads as described below (Philadelphia College of Osteopathic Medicine’s #A17-012). Enucleated eyes were fixed in 4% formaldehyde for three hours, washed in phosphate buffered saline (PBS) (Gibco; Thermo Fisher Scientific), embedded in paraffin and sectioned at 8 μM.

### Assays of phagocytosis in explant cultures of human lens tissue

Human anterior lens tissue was divided into two pieces. One piece was suspended in 300 μl DMEM/F12 medium (DF) containing 3 IU penicillin and 30 μg streptomycin (DF) (Gibco; Thermo Fisher Scientific). After 18 hours, the tissue was pressed at the edges onto a 35 mm tissue culture plastic dish and incubated in 50 μl of DF [13].

The other piece of lens tissue was suspended for 18 hours in 200 μl DF containing 35 μM doxorubicin (Sigma-Aldrich) in Lab-Tek Chamber slides (Thermo Fisher Scientific) to induce cell death [14]. The tissue was rinsed twice in DF, added to a fresh well containing the pH sensitive dye included in the IncuCyte pHrodo^™^ Red labeling kit for phagocytosis (Sartorius, Ann Arbor, MI), incubated for one hour and dissociated by repeated pipetting. Cells were rinsed twice by centrifuging and resuspending in DF. The cells were added to the untreated lens explant cultures in 500 μl of DF. The following day, cultures were fixed in 2% formaldehyde and labeled with the G8 mAb, a secondary antibody conjugated with Alexa 488 and Hoechst dye as described below.

A second assay was performed to demonstrate phagocytosis in the lens. Living Myo/Nog cells in the untreated piece of tissue were labeled with the G8 mAb and a fluorescent secondary antibody. Unbound antibodies were removed by rinsing. An hour later, cells from explants that had been treated with doxorubicin and incubated in pHRodo Red were added to the intact, pre-labeled explant. The cultures were fixed the following day and stained with Hoechst dye.

### Assay of phagocytosis of magnetic beads injected into the anterior chamber

Magnetic beads (4.5 μM Invitrogen Dynabeads^™^ M-450 Epoxy, Thermo Fisher Scientific) were injected at a concentration of 2.4 X 10^6^ in 2 μl of PBS using a 10 μl Gastight Hamilton syringe (Hamilton Company, Reno, NV) into the anterior chamber of 2-month old C57BL/6j mice (Jackson Laboratory, Bar Harbor, ME) according to the methods of Samsel et al. [18] and Ito et al. [19]. The glycidyl ether (epoxy) coating was removed from the beads prior to injection [19]. The beads were directed towards the iridocorneal angel using a magnet. The eyes were collected one month later.

### Immunofluorescence localization

Human lens explant tissue and tissue sections from human skin and rabbit and mouse eyes were examined for antibody binding as described previously [1, 3, 5]. A summary of the method is provided below. The numbers of animals, explants and tissue sections are given in Table 1.

**Table 1:** Tissue sources and numbers of tissue explants and sections screened by immunofluorescence localization of antibodies.

Tissue	# subjects/animals	Antibodies: # of tissue sections and lens explants analyzed
Human tattooed skin	6	G8: 8
		Noggin: 16
		G8/Noggin: 29
		G8/MyoD: 8
		G8/Iba1: 26
		Noggin/Iba1: 18
		G8/α-SMA: 32
		No primary antibody controls: 14
Human lens tissue	25	G8/pHRodo Red: 7
		G8/Iba1: 8
		No primary antibody controls: 10
Rabbit anterior cavity	16	G8/Iba1: 15
		No primary antibody controls: 5
Mouse eyes	5	G8: 40
		No primary antibody control: 4

Prior to applying antibodies, tissue and tissue sections were incubated in blocking buffer consisting of 10% goat serum in phosphate buffered saline (Gibco; Thermo Fisher Scientific). Incubations in primary and secondary antibodies diluted blocking buffer were carried out at room temperature in a humid chamber for 30 minutes. Myo/Nog cells were identified with antibodies to the G8 antigen and Noggin. The G8 IgM mAb was generated by immunizing BALB/c mice with whole, fixed cells isolated from the posterior 12 pairs of somites and segmental plate mesoderm of stages 12–14 chick embryos (5). G8 mAb culture supernatant was diluted 1:20 and purified G8 IgM was used at a concentration of 3.6 μg/ml. The anti-Noggin goat polyclonal antiserum (AF719; R&D Systems, Minneapolis, MN), anti-MyoD IgG1 mAb (MA5-12902, Thermo Fisher Scientific, Rockford, IL) and anti-MyoD rabbit polyclonal antiserum (ab203383, Abcam, Cambridge, MA) were diluted 1:100. The antibody to alpha smooth muscle actin (α-SMA) mAb was directly conjugated with fluorescein (F3777, Sigma-Aldrich, St. Louis, MO) and diluted 1:250. The anti-Iba1 rabbit mAb (ab178846, Abcam) and anti-Iba1 goat polyclonal antiserum (ab5076, Abcam) were diluted 1:100 and 1:50, respectively.

Explants and tissue sections were rinsed three times in PBS before incubating in AffiniPure Fab fragment subclass and species-specific secondary antibodies conjugated with Rhodamine Red and Alexa 488 (Jackson ImmunoResearch Laboratories, Inc., West Grove, PA) diluted 1:400. Explants and tissue sections were rinsed twice in PBS and once in deionized water then mounted in Fluoro-Gel with 4’,6-diamidino-2-phenlindole (DAPI) (Electron Microscopy Sciences, Hatfield, PA) or Vectashield mounting medium (Vector Laboratories, Burlimgame, CA). The level of background fluorescence was assessed by labeling tissue sections and explants with secondary antibodies only.

### Microscopy

Tissues were analyzed with the Nikon Eclipse E800 epifluorescent microscope (Nikon Instruments Inc., Melville, NY) equipped with the following lenses: 10x, numerical aperture (na) 0.45; 60x oil immersion, na 1.4; and 100x oil immersion, na 1.4. The Evolution QE Optronics video camera and Image Pro Plus image analysis software program (Media Cybernetics, Rockville MD). Sections also were analyzed with the Olympus Confocal Fluoview 100 microscope (Olympus Corp., Tokyo, Japan) equipped with a 60x oil immersion lens, na 1.4 and the Fluoview software program. Adobe Photoshop CC 2014 (Adobe Inc., San Jose, CA) was used to adjust photographs for brightness and contrast, and assembly and annotation of figures.

## Results

### Myo/Nog cells contain tattoo ink in human skin

**Fig 1:**
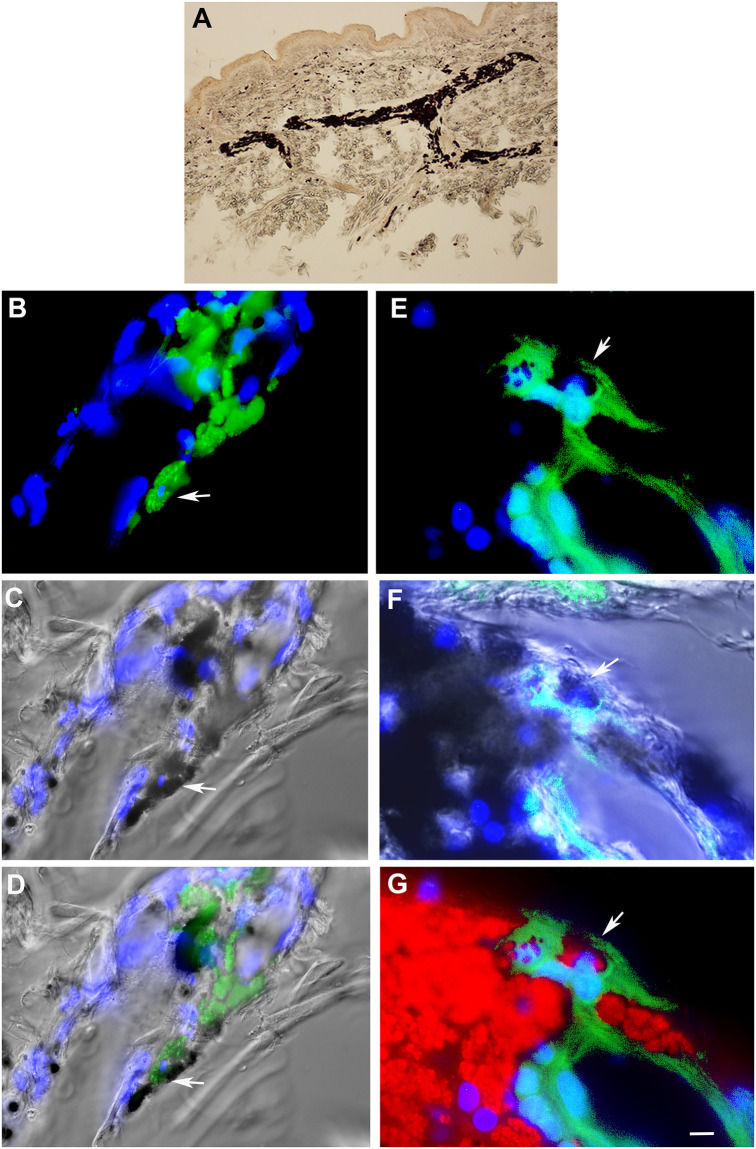
Myo/Nog cells contain ink in human tattooed skin. An unstained tissue section of tattooed skin is shown in A. Other sections were labeled with the G8 mAb (green) and Hoechst dye (blue). Confocal 60x, 0.5 μM step size images of the same field are shown in B-D. E-F were photographed with the 60x epifluorescent microscope. Green and blue photomicrographs were merged in B and E. Fluorescent photomicrographs were merged with the corresponding DIC image to visualize ink (C, D and F). Tattoo ink appears black in DIC images (C, D and F). Red ink fluoresces in the rhodamine channel of the epifluorescence microscope (G). Some G8+ Myo/Nog cells appeared to contain ink. Bar = 540 μM in A and 5.6 μM in B-G.

We previously reported that Myo/Nog cells expressing G8, Noggin and MyoD mRNA were associated with the hair follicles, rapidly responded to epidermal abrasion and home to skin tumors [20]. The potential of Myo/Nog cells to engage in phagocytosis was explored by examining tissue sections of tattooed human skin. Ink injected to create tattoos is internalized by phagocytosis and remains visible in cells, including fibroblasts and macrophages that have engulfed it [21–25]. Aggregates of Myo/Nog cells accumulated in tattooed tissue and some appeared to contain ink, as evidenced by the presence of G8, ink and a nucleus in the same 0.5 μM step size confocal section (Fig 1B–1D). Additional evidence of internalization of ink in Myo/Nog cells is illustrated in epifluorescence photographs in which red ink fluoresces in the cytoplasm of a G8+ cell (Fig 1E–1G).

**Fig 2:**
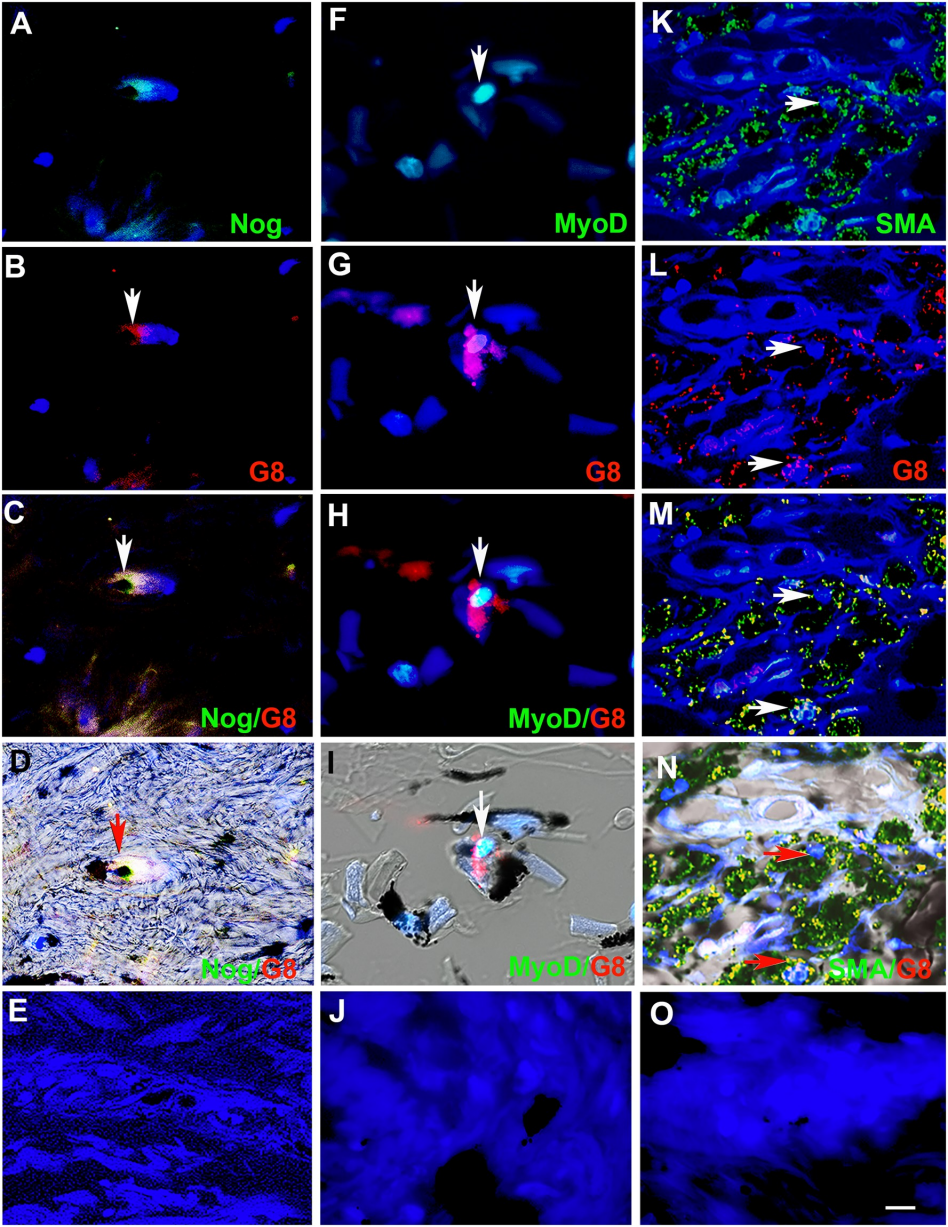
Myo/Nog cells contain ink in human tattooed skin. Tissue sections of tattooed skin were double labeled with the G8 mAb and antibodies to Noggin (Nog) (A-D), MyoD (F-I) or α-SMA (K-N). The colors of the secondary antibodies are indicated in the photographs. Nuclei were stained with Hoechst dye (blue). Images were produced with the epifluorescence (A-E, J and O) and confocal microscopes (F-I and K-N) with 60x lenses. Overlap of green and red fluorescence appears yellow in merged images (C, D, H, I, M and N). Fluorescent photomicrographs were merged with the corresponding DIC image to visualize the ink (black in D, I and N). Some double labeled cells appeared to contain ink (arrows in D, I and N). All ink laden G8+ cells were α-SMA+ (N). Smooth muscle cells of blood vessels also contained α-SMA (K). Minimal to no background fluorescence was visible after staining with the anti-goat (E), anti-IgM and anti-IgG (I), and anti-rabbit (O) secondary antibodies only. Bar = 28 μM in E and 5.6 μM in A-D and G-O.

All G8+ cells in the skin were labeled with the Noggin antibody and vice versa (Fig 2A–2D). A subpopulation of G8+ cells contained MyoD protein (Fig 2F–2I). Some G8+/Noggin+, G8+/MyoD+ and G8+/MyoD- cells appeared to contain ink (Fig 2D and 2I). Tissue sections of tattooed skin also were double labeled with antibodies to G8 and α-SMA to determine whether Myo/Nog cells with ink had differentiated into myofibroblasts. All G8+ cells that appeared to contain ink were α-SMA+ (Fig 2K–2N). More cells with ink were unlabeled with either antibody (Fig 2N). Labeling with the secondary antibodies produced minimal to no background fluorescence (Fig 2E, 2J and 2O).

### Myo/Nog cells in human lens tissue phagocytose dead cells

**Fig 3:**
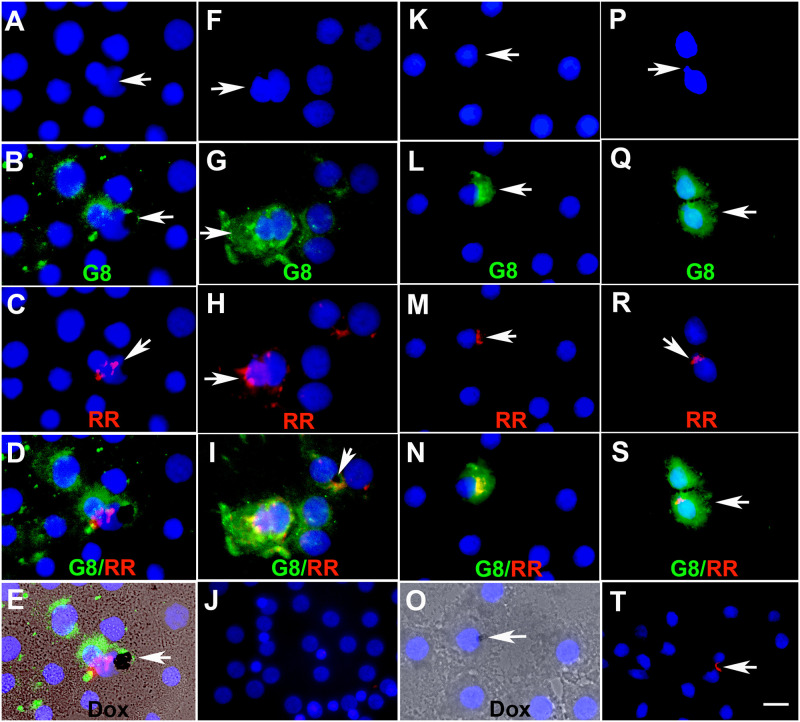
Phagocytosis of dead cells by Myo/Nog cells in human lens tissue. Anterior lens tissue removed during cataract surgery was treated with doxorubicin then incubated with pHrodo Red^™^ that fluoresces after phagocytosis and internalization into acidic compartments of cell. Cells were dissociated and added to untreated lens tissue. A-E and F-I are photographs of two cultures that were fixed and labeled with the G8 mAb (green) and Hoechst dye (blue) 18 hours after addition of treated cells. In K-O and P-S, living cells in untreated lens explants were labeled with the G8 mAb before adding cells from explants treated with doxorubicin and pHrodo Red. Tissue was imaged with the epifluorescence microscope and the 100x (A-I and K-S) or 60x lens (J and T). Unmerged images of nuclei are shown in A, F, K and P. Arrows demonstrate the presence of two nuclei (A and F) or nuclear remnants (P) in a single cell. Merged images of G8 and Hoechst, and pHrodo Red and Hoechst (arrows) are shown in B, G, L and Q, and C, H, M and R, respectively. D, I, N and S are triple merges of blue, green and red. Overlap of red and green appears yellow. Doxorubicin appears black in the quadruple merge of the three fluorochromes and DIC in E, triple merge in I and Hoechst staining and DIC in O (arrows). Some G8+ contained two nuclei or nuclear remnants, pHrodo Red fluorescence (A, I, N and S) and doxorubicin (E and O), indicating phagocytosis. Control cultures labeled with only the secondary antibody and Hoechst dye before (J) or after addition of treated cells (T) illustrate the absence of background green fluorescence. pHrodo Red is visible at the arrow in T. Bar = 9 μm in J and Tand 15 μm in A-I and K-S.

Myo/Nog cells are attracted to dying cells in the blastocyst, rabbit lens and murine retina [7, 10, 16]. They also home to wounds in anterior human lens tissue removed during cataract surgery [13, 14]. Determination of whether Myo/Nog cells engage in phagocytosis in human lens tissue was carried out by inducing cell death with doxorubicin [14] and labeling with pHrodo Red^™^ that binds to the cell surface, does not diffuse into the cytoplasm and fluoresces after it is phagocytosed and internalized into acidic compartments of the cell [26]. Red fluorescence was not observed in explants incubated with pHrodo Red without previous treatment with doxorubicin. Treated cells were added to untreated lens explants. The following day, cells were labeled with the G8 mAb. Some G8+ cells contained fluorescent pHrodo Red (Fig 3A–3I). Two nuclei were present in a subpopulation of G8+/pHrodo Red+ cells (Fig 3A and 3F). Doxorubicin was visible in some of these cells (Fig 3E). G8+ and G8- cells surrounding phagocytosed cells did not fluoresce with pHrodo Red (Fig 3A–3I).

Similar results were obtained when living cells in the untreated piece of lens tissue were labeled with G8 and secondary antibody prior to adding cells from the explant that had been incubated with doxorubicin and pHrodo Red (Fig 3K–3S). This control experiment demonstrates that G8+ Myo/Nog cells present in the untreated explant had phagocytosed dead cells added to their culture. Minimal red autofluorescence or background from the anti-IgM secondary antibody was visible in the explants (Fig 3J and 3T).

### Myo/Nog cells engulf beads injected into the anterior chamber of the eye

**Fig 4:**
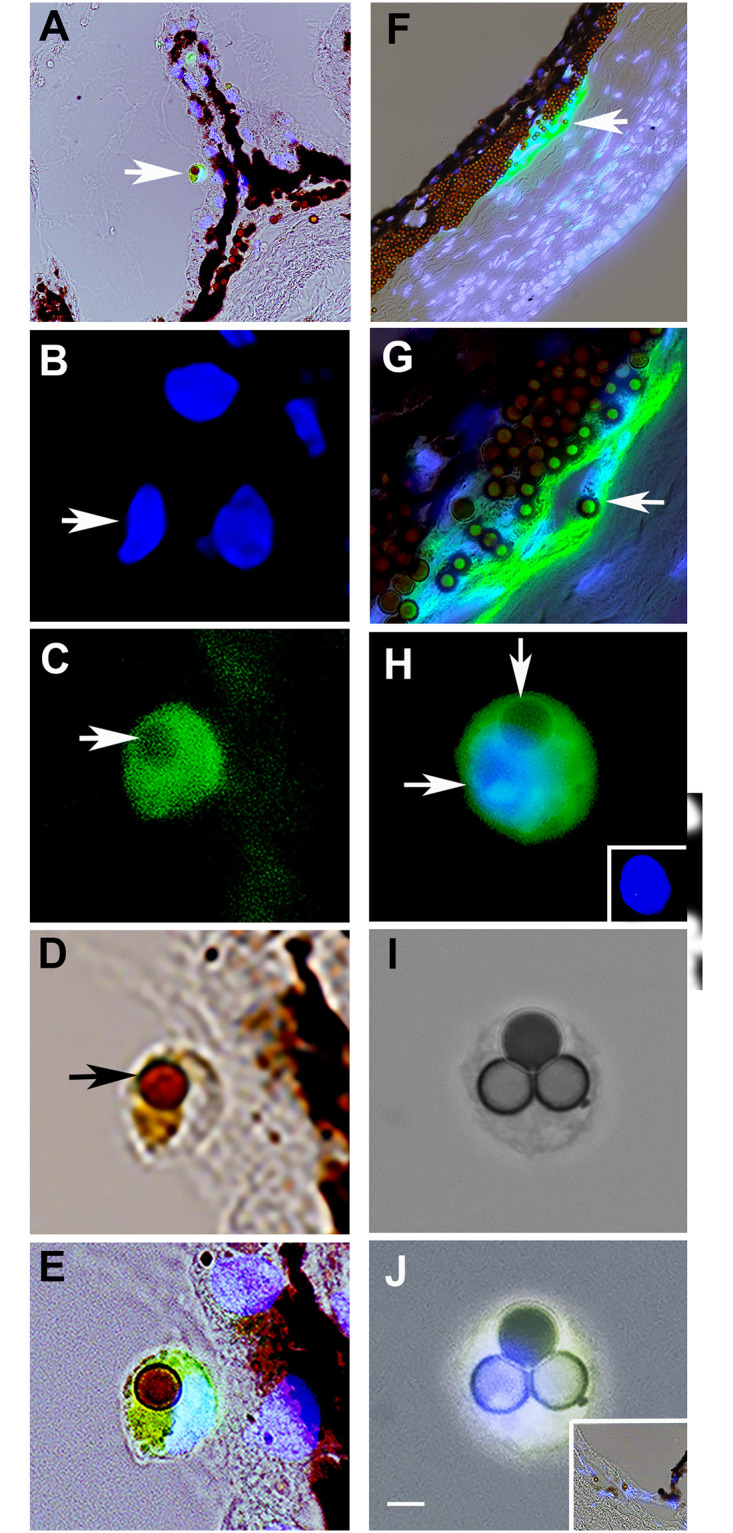
Engulfment of beads by Myo/Nog cells in the anterior cavity. Beads were injected into the anterior chamber of mice and drawn to the iridocorneal angel with a magnet. One month later, tissue sections were labeled with Hoechst dye (blue) and the G8 mAb (green) and imaged with the epifluorescent microscope and 60x lens. A-E, F and G, and H-J are photographs of bead laden cells on the surface of a ciliary process, within the iridocorneal angle and between the ciliary body and lens, respectively. Cells at the arrows in the merged images of fluorescence and DIC in A and F are shown in B-E and G, respectively. Pigment appears black when photographed with DIC optics (A, D, E and F). Beads (brown/gray) are visible in the DIC images (D and I) and triple merges of DIC, Hoechst dye and G8 (A, E, F, G and J). Arrows point to nuclei in B and H and beads in C, D, G and H. The inset in H shows the Hoechst staining of the nucleus. The inset in J is a section of the ciliary body labeled with the anti-IgM secondary antibody only. Beads are present in the cytoplasm of G8+ Myo/Nog cells. Bar = 9 μm A and F and 2.5 μm in B-E and G-J.

Injection of microbeads into the anterior chamber induces glaucoma in mice [18]. Labeling with the G8 mAb revealed the presence of Myo/Nog cells in areas of bead accumulation within the tissue of the iridocorneal angle (Fig 4F and 4G). Beads were definitively resolved within the cytoplasm of G8+ cells on the surface of the ciliary body (Fig 4A–4E) and between the ciliary body and lens (Fig 4H–4J) in the anterior cavity. One Myo/Nog cell contained three beads (Fig 4H–4J). This experiment revealed that Myo/Nog cells are capable of engulfing magnetic beads in the anterior cavity of the eye.

### Myo/Nog cells are distinct from macrophages in the skin and anterior cavity of the eyes

**Fig 5:**
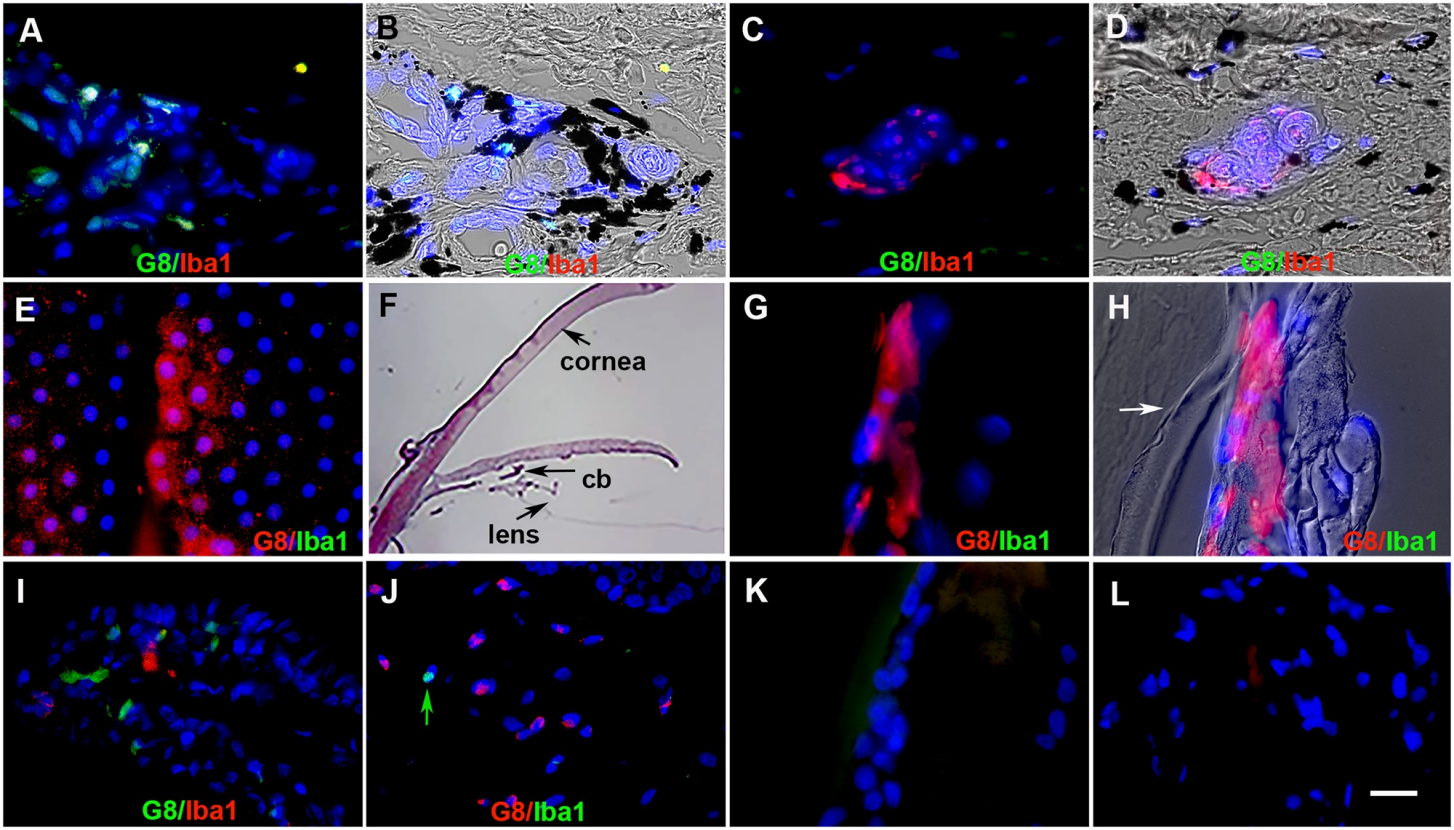
The G8 and Iba1 mAbs label separate populations of cells in the skin and anterior cavity of the eye. Tissue sections of human tattooed skin (A-D), human lens tissue (E) and sections of the rabbit eye two days after cataract surgery (G-L) were double labeled with the G8 and Iba1 mAbs and imaged with the epifluorescent microscope and 60x lens. The colors of the secondary antibodies are indicated in the photographs. A section through the anterior cavity was stained with hematoxylin and eosin (F). Photographs in B, D and H are quadruple merges of DIC showing black ink and blue, red or green fluorescence. G8+ cells lacked staining for Iba1 in the skin (A and B). Iba1+/G8- cells are shown in a different field in C and D. G8+ cells were present in the human (E) and rabbit lens (G and H), but no Iba1+ cells were detected in these tissues. The arrow in H illustrates the lens capsule. The G8 and Iba1 mAbs labeled separate subpopulations of cells in the ciliary body (cb) (I) and cornea (J). The arrow in J points to an Iba1+ cell. Minimal fluorescence was present in sections of the lens (K) and ciliary body (L) labeled with secondary antibodies only. Bar = 9 μM in A-E and G-L, and 270 μM in F.

We previously showed that Myo/Nog cells in skin tumors and the retina do not express the macrophage markers F4/80 and ionized calcium binding adaptor molecule 1 (Iba1), respectively [10, 20]. For our analyses of tattooed human skin, we paired the G8 and Iba1 mAbs to determine whether Myo/Nog cells containing ink were related to macrophages and if macrophages had also phagocytosed ink. The two mAbs labeled separate populations of cells (Fig 5A–5D). There also was no overlap in staining with the Noggin and Iba1 mAbs (data not shown). Subpopulations of G8+/Iba1- and G8-/Iba1+ cells contained ink (Fig 5B and 5D). In all sections of the skin, the nuclei of Myo/Nog cells were round or oval shaped and lacked segmentation typical of granulocytes (Figs 1, 2 and 5). These results indicate that Myo/Nog cells in human skin are distinct from macrophages and granulocytes, and both macrophages and Myo/Nog cells in the skin had phagocytosed tattoo ink.

Double labeling with the G8 and Iba1 mAbs also was performed to examine the relationship between Myo/Nog cells and macrophages in the anterior cavity of the eye (Fig 5). G8+ cells were present throughout the anterior cavity (Fig 5E–5J), as observed previously [13, 15, 16]. No Iba1+ cells were present in human lens explant cultures (Fig 5E). In tissue sections of the anterior cavity of rabbits that had undergone cataract surgery, G8+ were observed in the lens, ciliary body and cornea, but they did not bind the Iba1 mAb (Fig 5G–5J). Iba+/G8- cells were present in the ciliary body and cornea (Fig 5I and 5J) but not the lens (Fig 5G and 5H).

## Discussion

The process of phagocytosis is essential for clearance of dead cells, microorganisms and other foreign material [27]. Lack of clearance of apoptotic cells may lead to necrosis, autoimmunity and chronic inflammation [28–31]. Three categories of phagocytic cells have been described [32]. Professional phagocytes are innate immune cells, including neutrophils, dendritic cells and macrophages, whose main function is engulfment. Specialized phagocytes, such as the retinal pigmented epithelium and sertoli cells of the testis, ingest apoptotic cells and cellular debris while maintaining tissue barriers and supporting surrounding cells. Choroidal cells and astrocytes of the central nervous system also have features of specialized phagocytes. The third category consists of nonprofessional phagocytes, such as smooth muscle cells and fibroblasts, whose primary functions are unrelated to phagocytosis but are capable of engulfment [33, 34].

Myo/Nog cells appear to fall in the category of nonprofessional phagocytes because they are distinct from macrophages and neutrophils, and do not form an epithelium (Figs 1–5) [2, 3, 9–11, 13]. They also do not have a barrier function, although aggregates of Myo/Nog cell-derived myofibroblasts may physically restrict the movement of other cells in the lens [13, 14, 16]. The primary functions of Myo/Nog cells are to: 1) regulate BMP signaling via the release of Noggin; 2) protect neurons in the retina; 3) home to wounds; and 4) differentiate into contractile myofibroblasts [3, 7, 10–16].

While future studies will define the overall contribution of Myo/Nog cells to clearance, the experiments described herein provide evidence that phagocytosis is a component of Myo/Nog cells’ response to perturbations in homeostasis resulting from injecting tattoo ink into the skin, cell death in the lens and addition of magnetic beads to the anterior chamber. In the skin, Myo/Nog cells remained within the injected area after appearing to internalizing ink, thereby contributing to the permanence of the tattoo. It also is possible that Myo/Nog cells had engulfed cells originally responsible for ingesting ink. This study did not reveal whether Myo/Nog cells or their differentiated myofibroblast progeny internalized ink; however, all G8+ cells laden with ink were α-SMA+.

Myofibroblasts engaging in phagocytosis have been described in the diseased heart and basal cell epitheliomas [35, 36]. In the failing heart, myofibroblasts were proposed to differentiate from bone marrow-derived cells, pericytes, cells that had undergone an epithelial-mesenchymal transition and resident fibroblasts [36–38]. Myo/Nog cells are present in the embryonic [4] and adult heart (unpublished data), as well as in tumors [9, 17], and therefore, they may contribute to the population of phagocytic myofibroblasts described previously.

Detection of pH Rodo red fluorescence in G8+ cells provides evidence that Myo/Nog cells engulfed apoptotic cells in explants of human lens tissue. The mechanism whereby Myo/Nog cells internalize cell corpses may involve brain-specific angiogenesis inhibitor 1 (BAI1), a cell surface receptor for phosphatidylserine that appears on the outer leaflet of the plasma membrane of dying cells [28, 39, 40]. BAI1 is expressed by Myo/Nog cells in the lens and other tissues [41].

Myo/Nog cells’ apparent engulfment of tattoo ink and internalization of magnetic beads, in addition to apoptotic cells, suggests that they employ multiple mechanisms to recognize and internalize foreign material. Ink may be a mixture of both organic and inorganic molecules, in part depending on the age of the tattoo [42]. The magnetic beads used in this study were washed to remove the organic epoxy coating surrounding iron oxide. In addition to engagement of BAI1, “eat me” signals may be propagated following binding to scavenger, Fc and complement receptors, cholesterol, lectins and annexin, and via additional undefined pathways [43–45].

Macrophages are professional phagocytes that engulf apoptotic and necrotic cells, microorganisms and organic and inorganic compounds [43–45]. The macrophage markers Iba1 and F4/80 were not detected in Myo/Nog cells [10, 20]. Expression of MyoD and Noggin also distinguish Myo/Nog cells from macrophages [1, 2]. Potential interactions between the two cells types and similarities and differences in molecular pathways regulating their attraction to and engulfment of foreign materials are important areas for future study.
